# Shape Memory Alloys for Aerospace, Recent Developments, and New Applications: A Short Review

**DOI:** 10.3390/ma13081856

**Published:** 2020-04-15

**Authors:** Girolamo Costanza, Maria Elisa Tata

**Affiliations:** 1 Industrial Engineering Department, University of Rome Tor Vergata, Via del Politecnico 1, 00133 Rome, Italy; 2 Civil Engineering and Computer Science Department, University of Rome Tor Vergata, Via del Politecnico 1, 00133 Rome, Italy; elisa.tata@uniroma2.it

**Keywords:** shape memory alloys, nitinol, smart structures, aerospace applications, intelligent systems

## Abstract

Shape memory alloys (SMAs) show a particular behavior that is the ability to recuperate the original shape while heating above specific critical temperatures (shape memory effect) or to withstand high deformations recoverable while unloading (pseudoelasticity). In many cases the SMAs play the actuator’s role. Starting from the origin of the shape memory effect, the mechanical properties of these alloys are illustrated. This paper presents a review of SMAs applications in the aerospace field with particular emphasis on morphing wings (experimental and modeling), tailoring of the orientation and inlet geometry of many propulsion system, variable geometry chevron for thrust and noise optimization, and more in general reduction of power consumption. Space applications are described too: to isolate the micro-vibrations, for low-shock release devices and self-deployable solar sails. Novel configurations and devices are highlighted too.

## 1. Introduction

**Figure 1:**
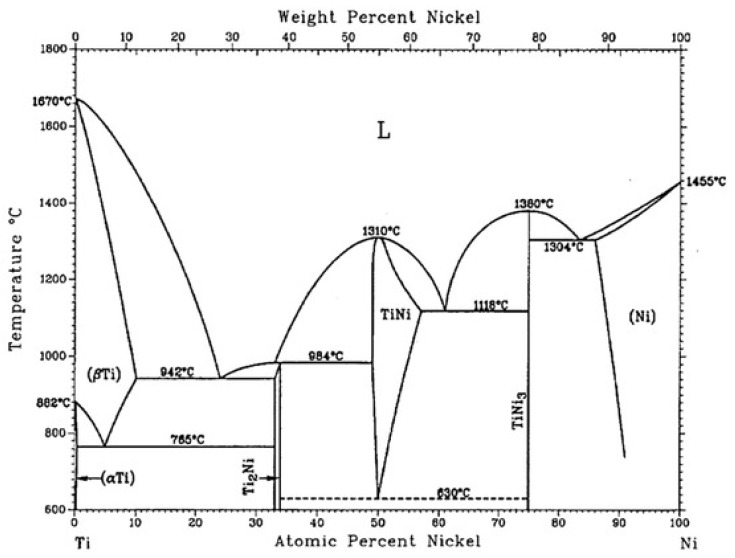
Binary phase diagram of Ti-Ni alloy [7]. Figure 1 Reprinted from Materials, Vol. 12 (5), Chekotu, J.C.; Groarke, R.; O’Toole, K., Brabazon, D., Advances in Selective Laser Melting of Nitinol Shape Memory Alloy Part Production, 2019.

Shape memory alloys (SMAs) are metals able to recuperate a preset shape just while heating up to a critical transformation temperature. The physical principle they are based on is a particular martensitic transformation (thermoelastic) occurring in some metallic alloys [1]. The list includes but is not limited to NiTi, CuAlNi, CuZnAl, AuCd, and many others. Buehler et al. in 1963 [2] were the first to evidence the shape memory effect in near equiatomic Ti-Ni alloys. The shape memory effect was found earlier in Au-47.5% Cd [3] and In-Tl [4,5] but without attracting attention from the researchers. On the contrary, equiatomic Ni-Ti alloys became popular thanks to Naval Ordinance Laboratory, hence the name Nitinol. The real understanding of the phenomena occurring during the martensitic transformation was not immediate. The main reason may be ascribed to the complexity of the Ti-Ni phase diagram (Figure 1) and to the formation of a multiplicity of precipitates under certain heat-treatments (TiNi, Ti_2_Ni, TiNi_3_, Ti_2_Ni_3_, Ti_3_Ni_4_). Moreover, the R-phase (rhombohedral) transformation, considered to be a pre-martensitic phenomenon, appears under certain conditions while cooling. Now it is established that the R-phase itself is a martensitic transformation and contends with the successive martensitic transformation. Due to the excellent mechanical properties, corrosion, and abrasion resistance Ni-Ti shape memory alloys have been widely employed in many technological applications: stent, guide wire, antenna for mobile phones, coffee maker, orthodontic wire, flap for air-conditioner, and many others. The main aim of this review is to identify and exemplify a detailed list of novel applications of SMA in the aerospace field in the most updated manner without the claim of comprehensiveness, according to available recent literature in terms of relevance, number of articles, and novelty of the proposals. Ni-Ti alloys are the most employed ones, but the subject is not limited to them.

## 2. Origin of the Shape Memory Effect

For the comprehension of the basic principles regarding the shape memory effect and pseudoelasticity, many contributions are useful. The crystallography of martensite, the transformation temperatures, and rate of martensite formation are discussed in details by Nishiyama [6].

The macrostructure consequences of the microstructure and the shape memory effect have been highlighted by Bhattacharya [8]. The design of some shape memory alloy actuators were focused on by Otsuka and Wayman [9]. National Aeronautics and Space Administration (NASA) report SP5111 published in 1972 provides experimental results, although the base mechanism was not fully understood at that time [10]. The phase transformation occurring on an SMA is peculiar because it coexists with great strain and is fully recoverable, so that in a uniaxial tensile test in NiTi it can reach also 10% [1]. For this reason, SMAs belong to the family of the “active materials” [11]. SMAs can also provide high forces and wide displacements when activated, if compared to other active materials. Novel application fields continue to be identified [12]. Biomedical industry is the most served one, together with the aerospace industry. SMAs can be identified also into the class of the “smart materials”: an SMA component, with structural or functional purpose, can make a system simpler in comparison with the same manufactured piece adopting conventional know-how (for instance electromechanical or hydraulic). High activation loads and displacements generates great energy density. These properties are welcome especially in the aerospace field, hence the main reason of this review. The technological requirements of the aerospace field are more important than any considerations about the technology cost and this aspect can be identified as the real reason of the success of SMAs.

## 3. SMA Behavior

**Figure 2:**
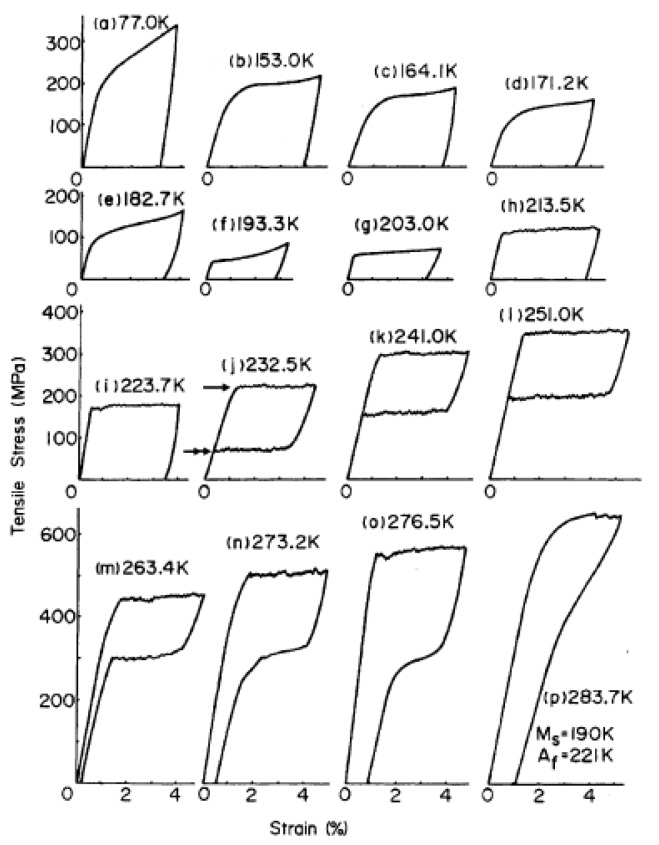
Stress-strain curves for a Ti-50.6Ni alloy showing shape memory effect (**a**–**i**) and pseudoelastic effect (**j**–**p**) [13]. Figure 2 Reprinted from Scripta Metallurgica, Vol. 15, Miyazaki, S.; Otsuka, K.; Suzuki, Y., Transformation pseudoelasticity and deformation behavior in a Ti-50.6Ni alloy, 1981, with permission from Elsevier.

In an extremely simplified manner, the main phases involved in an SMA are being austenite stable at higher temperature (parent phase) and martensite stable at lower temperature with tetragonal or monocline structure [1,8,12]. A lot of stress-strain curves obtained in tensile tests for the Ti-50.6Ni alloy at different and constant temperatures are reported in Figure 2 [13], highlighting the mechanical behavior from low temperature to high temperature. At lower temperatures (Figure 2a–i), it is evident the shape memory effect: after an initial linear stage a plateau of stress is evident, followed by residual strain while unloading. After that, the shape recovery (not shown in the graph) on heating starts at As (Austenite start temperature) and finishes at Af (Austenite finish temperature). At temperatures higher than Af (Figure 2j–p), austenite is the only stable phase. Tensile behavior exhibits a loading path with a plateau at higher stress than that in the martensitic phase. During unloading, the strain is fully recovered and the classic flag-type diagram is evidenced, without the necessity of further heating. The higher the temperature, the higher the level of stress for the plateau.

The aerospace industry is actively looking for novel solutions and applications based on the integration of the SMAs in the actual technologies as well as the definition and development of new ones. SMA adoption allows to increase the simplicity of the systems as well as to reduce the weight and the volume of such active devices allowing it to achieve more compact structures. SMAs are attractive as a solution to complex engineering problems, along with high actuation stresses and strains due to their intrinsic great power/weight ratio. Integrating actuators and structures means it is possible to achieve high reliability and compact arrangements, reliable also for a high number of activation cycles [14,15]. Systems activated by SMAs have been widely examined in many studies and pieces of research in substitution of standard actuation systems, hydraulic and electric.

## 4. SMA Applications on Wings

**Figure 3:**
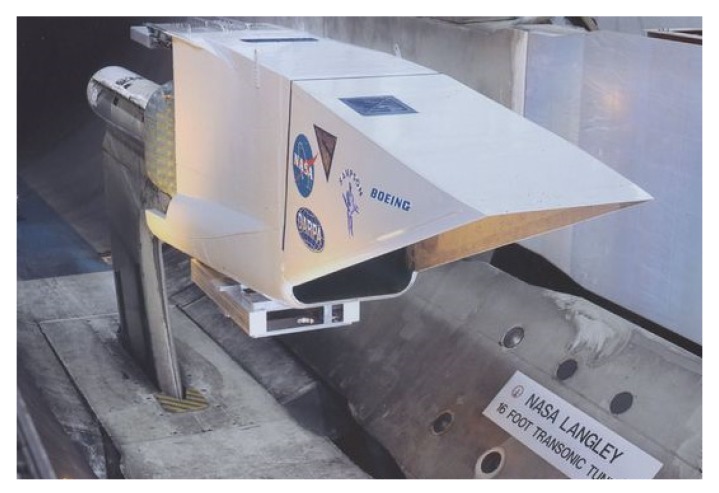
The SAMPSON F-15 inlet tested in the facility at Langley (NASA) [19]. Figure 3 Available online: https://crgis.ndc.nasa.gov/historic/File:2000-L-00475.jpg.

**Figure 4:**
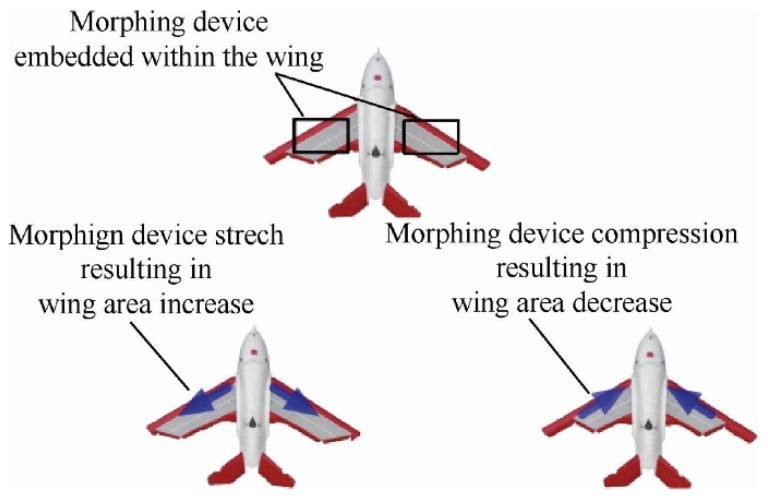
Various morphing devices employed on the wing airplanes can change, stretching or compressing, the aerodynamic profile [20]. Figure 4 Reprinted from Chinese Journal of Aeronautics, Vol. 32 (4), Chen, Y.; Shen, X; Li, J.; Chen, J., Nonlinear hysteresis identification and compensation based on the discrete Preisach model of an aircraft morphing wing device manipulated by an SMA actuator, 2019, open access.

Wing morphing in planes is a practical solution involving structural modifications so that the required specifications can be met in different utilization conditions. Some of the advantages are reported in the following: increased speed, reduced power consumption, roll control, and camber change. On the other hand, morphing is usually associated to drawbacks like surface discontinuity and being over-weight due to the necessary control structures. Adopting shape memory solutions and their corresponding mechanisms means some of these drawbacks can be overcome. Smart Wing Program and Smart and Aircraft and Marine Propulsion System Demonstration (SAMPSON) [16,17] are the most famous projects regarding fixed wings both recognized by Defense Research Projects Agency (DARPA). The first one was conceived for the utilization of smart materials like lifting devices thanks to the specific power and power density of SMA actuators in comparison with traditional electromechanical ones. The second one had a main goal of the development of new affordable smart materials and is concerned with shape-adaptive structures for aircraft. In particular, the Sampson project examined models for changing the shape in gas turbine inlets for application in supersonic airplanes. A novel application for a hydrodynamic maneuvering system to be employed in maritime propulsion has been investigated too. The shape memory effect has been employed to provide actuation by means of shape recovery [18,19]. The main goal was to show the possibility of adoption of SMAs in modifying the orientation and geometry in different propulsion devices. An experiment on the real F-15 inlet was performed in the configuration of an SMA cable antagonist to another one (Figure 3). Some examples of morphing wings are reported in Figure 4.

The F15E Strike Eagle is a fighter jet conceived to accomplish different kinds of missions. Thanks to the two engines, the aircraft can reach speeds higher than Mach 2.5. The inlet system of this jet consists of five ramps adopted for the conditioning of the air flow for both engines. The ramps are connected to a cowl which can rotate up to 15° as a function of the different flight conditions. The SMAs are employed to rotate the inlet cowl in order to change the air flow cross section. In the tested model, many modifications have been applied from the original configuration allowing to host the shape changing elements. A force of 26,700 N and 9° rotation angle have been applied by means of a bundles of 34 wires [21]. On the aft side of the fan nozzle shape, memory cables have been disposed circumferentially around and tested too [22] so that fan nozzle area can be modified in different operative conditions during flight. For the wind tunnel tests, the configuration with a sting attached in the lower side of the inlet by means of a plate support was adopted. For the experimental validation of the F-15 inlet, the NASA Langley wind tunnel was used (Figure 3). Slow SMA cooling and longer retraction time was the prime limitations of the proposed system, and even at the stage of experimental tests.

**Figure 5:**
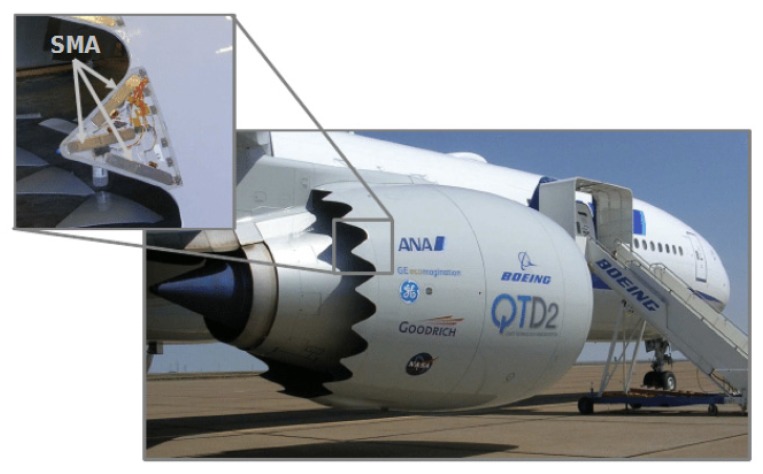
Variable geometry chevron activated by SMA in the full scale test [23]. Figure 5 Reprinted from Proceedings of the Institution of the Mechanical Engineer, Part. G: Journal of Aerospace Engineering, Vol. 221, Hartl, D.J.; Lagoudas, D.C., Aerospace applications of shape memory alloys, 535–552, 2007, open access.

A project to decrease noise at airports generated by departing airplanes was focused on by Hartl and Lagouda [23,24]. It has been demonstrated that the adoption of aerodynamic devices, better known as chevrons, on the rear edge of an exhaust nozzle (both on primary and secondary exhaust flow) can reduce jet noise at take-off thanks to the appropriate streams mixing. As a consequence of the secondary exhaust nozzle, chevrons immersed in the fan flow show a reduction of thrust and an increase of drag while cruising was detected. For these reasons, the optimum solution was identified in the Variable Geometry Chevron (VGC). Based on SMA components, VGC exhibits all-out bending during take-off and landing with minimum deflection flow while cruising so that efficiency is increased and noise reduction ensured. The system contains active SMA bundles enclosed in a complex case. The activation of the SMA beams allows the requested bending force on the chevron structure so that noise can be reduced. Boeing tested in flight the proposed solution adopting active SMA elements (Figure 5). At the end of the study results from experiments and modeling have been compared. Tip displacement and chevron deflection have been analyzed focusing the attention on the actuation profile. The developed model can be a useful instrument for the prediction of mechanical actuation of such a system subjected to pre-determined thermal inputs. Suitable systems for use in service are still under investigation.

In addition to the propulsion system, SMAs can be usefully applied in adapting lifting bodies and morphing the wing structures. The integration of the SMA elements in aerostructure has been focused in many studies. One of the promising approaches is to insert SMA wires into an innovative composite structure. In order to exploit the one-way shape memory effect, NiTi alloy wires of 150 μm diameter have been pre-stressed and inserted into a Kevlar fiber epoxy matrix [25]. SMA composites have a great potential in adaptive uses such as progressive reinforcing of components (structure) or change of the intrinsic vibration frequencies. However, the SMA behavior is not linear and offers many options. Moreover, increased knowledge regarding the stress transfer between metal and polymer matrix is required as well the fatigue behavior of such structures. Another application concerns SMA wire actuators, which can be connected to some internal points of an airfoil and activated to change the shape of the airfoil itself. The change in shape can rise the wing’s efficiency also in different flow regimes in operative flying conditions. The main goal to identify the number and the right positioning in the wing of the SMA wire actuators within the wing through a global optimization method was achieved. For the verification of the predicted structural and aerodynamic response, a suitable wing model to be tested in a wind tunnel was manufactured. The numerical pressure data have been found to be in good agreement with experiments performed in the wind tunnel tests, according to which a higher value of lift for a fixed speed and angle of attack by activating the SMA wire actuators was detected [26].

**Figure 6:**
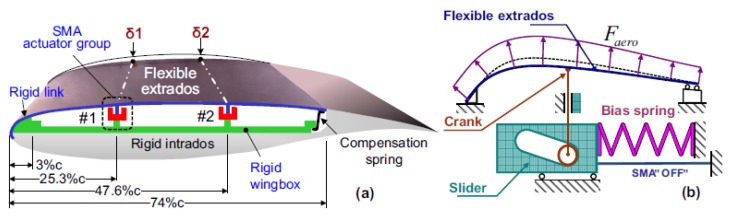
(**a**) Sketch of the morphing laminar wing adopting extrados with shape memory alloy (SMA) active element [27]. (**b**) Scheme of the SMA actuator’s working principle [27]. Figure 6 Reprinted from Physics Procedia, Vol. 10, Brailovski, V.; Terriault, P.; Georges, T.; Coutu, D., SMA Actuators for morphing wings, 197–203, 2010, open access.

SMA actuators to be employed for morphing wings attracted researchers’ attention. Brailowski et al. [27] modeled morphing wings by means of a coupled thermo-mechanical approach and the results have been validated in some tunnel tests. An experimental Morphing Laminar Wing (MLW) was set-up to demonstrate the practicability of airplanes’ fuel saving by improving the laminar flow regime on the wing extrados while cruising (subsonic motion). The prototype combined three main subsystems: flexible extrados, rigid extrados, and actuator positioned in the wing box (Figure 6). The wing morphing capacity was based on precise buckle of the wing extrados obtained by means of SMA actuators. The evaluation of the mechanical and aerodynamic behavior of the morphing wing in various flight scenario was possible thanks to a coupled model (fluid and structure). A small-scale morphing wing prototype (chord 0.5 m, span 1 m) was tested in a tunnel wing under subsonic conditions. The integration of the coupled thermomechanical finite element model with the previously developed structure-aerodynamic one of the morphing wing can be successfully employed to achieve the optimum solution of the whole system.

Coutu et al. [28] identified two core design constraints: the number of layers in the composite of the flexible extrados and the number of actuators. In order to equipoise the trade-off between stiffness and elasticity of the whole active structure, mechanical (deformation energy) and aerodynamic (laminar air flow regime improvement) performance benchmarks have been taken into account at the same time. By means of the multi-objective optimization practice, the designers selected the configuration 4-ply 2-actuator for the extrados configuration activated by SMA elements.

A method based on the discontinuous Preisach modeling, able to identify the hysteretic behavior of the morphing wing, has been suggested by Chen et al. in [20]. A group of discontinuous and equidistant points was applied to the Preisach plane so that the Preisach density in the selected unit was determined. Discontinuous Preisach modeling is achieved by the discontinuous first-order reversible curve. Thanks to the discrete Preisach model, it has been possible to simulate the airplane morphing wing; the effectiveness of the adopted model has been shown by opposing the modelled results with experimental results of the main hysteresis and the wing displacement. Afterwards, a hysteresis compensation strategy was defined for the actuation and control of the airplane wing. Such a device manifested a hysteretic behavior in which the shrinking had been compensated. In this way it has been possible to achieve the extension of the morphing application also to the intrinsic hysteretic region.

Another interesting advantage of this technical solution is that morphing wings can generate smoother wing surfaces, thus increasing airplane agility and efficiency by adopting discontinuous moving surfaces. A novel solution based on a double corrugated variable camber structure with morphing units of the rear edge has been proposed by Kumar et al. [29]. Two configurations have been considered: the first one is thought on the basis of a fish bone active camber, the second one on an adjustable camber morphing wing made of single corrugated structure. The comparison between the two configurations has been performed in terms of structural and dynamic analysis. The structural investigation of all simulations was carried out by means of the finite element analysis with particular reference to the actuation mechanism. The aerodynamic analysis was performed following two different techniques: the analytical approach based on thin airfoil theory and the other one numerically using Xfoil software. The last one combines a potential-flow panel process with viscous boundary-layer solver. An assessment was performed according to the stress and strain established in different portions of the models. The results have evidenced that the configuration double-corrugated variable camber morphing arrangement was able to undertake a higher level of loads in absence of detrimental strain. On the other hand, the aerodynamic investigation has shown that the efficiency of such a morphing wing is greater in comparison with other configurations.

An interesting historical review about morphing aircraft can be found in [30]. The challenge to concept a structural element that is able not only to allow the prescribed loads but with a shape adaptable to the various operative conditions has been well illustrated. The right mixing of morphing structures and smart actuators in the perspective of an integrated approach requires multidisciplinary thinking, from the initial development at the preliminary design stage. The review presents the state-of-the-art on morphing airplanes. Attention is focused on the fundamental concepts and SMA-activated morphing wing for both configurations (fixed and rotary wings) and in particular to the active systems. Solutions based on inflatable devices and skin issues have not been considered. In general, the penalty due to the weight increase associated with the actuation systems in any successful wing morphing system must be overcome. Despite that many fascinating concepts have been combined, just a few have reached the development stage for the wind tunnel test and ever fewer have been tested in real flight conditions.

**Figure 7:**
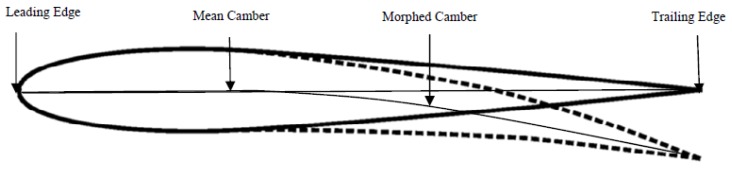
Camber change of activated and deactivated airfoil profile [31]. Figure 7 Reprinted from Materials Today Proceedings, Vol. 5, Hattalli, V.L.; Sritvasa, S.R., Wing morphing to improve control performance of an aircraft—An overview and a case study, 21442–21451, 2018, with permission from Elsevier.

An interesting overview and a case study of morphing wing for the improvement of the control performance of airplanes have been illustrated by Hattalli and Srivatsa [31]. The case study focused on the camber change of the horizontal tail of an aircraft employing SMAs. A numerical analysis was carried out aimed at the examination of the camber change by means of shape memory actuators. A maximum deflection of 0.6 mm at the trailing edge tip (Figure 7) on the horizontal tail yielded was detected. By modulating the shape memory intensity and the positioning of the strips, a novel and enhanced model for the delivery of a morphable wing was defined.

The comparison of the results from wind tunnel tests and numerical modeling regarding a morphing wing fitted out with flexible upper surface actuated by aileron have been presented by Gabor et al. [32]. The experimental setup was demonstrative of a real airplane’s wing tip section. The development was performed according to a multifaceted and multidisciplinary design process. The prototype was tailored by a composite material skin on top with a morphable shape according to the various flight conditions exploiting four shape memory devices positioned inside the wing. The main aim of the optimization was to control the level of the region under laminar flow. The subsequent shapes were acquired by means of an accurate photogrammetry technique. Computational fluid dynamics analysis was conducted and a suitable model for the prediction of the transition from laminar to turbulent flow on the wing surface was developed. Three different aileron deflection angles and angles of attack in the range of five degrees were considered. The outcome of the simulation was compared with the measurements carried out by means of infrared thermography technique. In this case, during tunnel testing in subsonic conditions surface pressure measurements, balance load measurement and transition location were acquired. Good agreement was found between numerical and infrared thermography results with a 5% average deviation of the chord.

**Figure 8:**
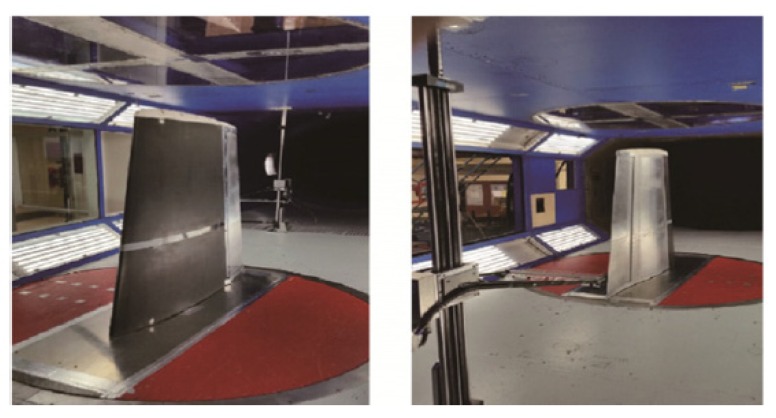
Wing model setup in wind tunnel section [34]: Leading edge (**Left**); Trailing edge (**Right**). Figure 8 Reprinted from Chinese Journal of Aeronautics, Vol. 30 (1), Koreanski, A.; Gabor, O.S.; Acotto, J.; Brianchon, G.; Portier, G., Botez, R.M.; Mamou, M.; Mebarki, Y., Optimization and design of an aircraft’s morphing wing-tip demonstrator for drag reduction at low speed, Part. II – Experimental validation using Infra-red transition measurement from wind tunnel tests, 164–174, 2017, open access.

**Figure 9:**
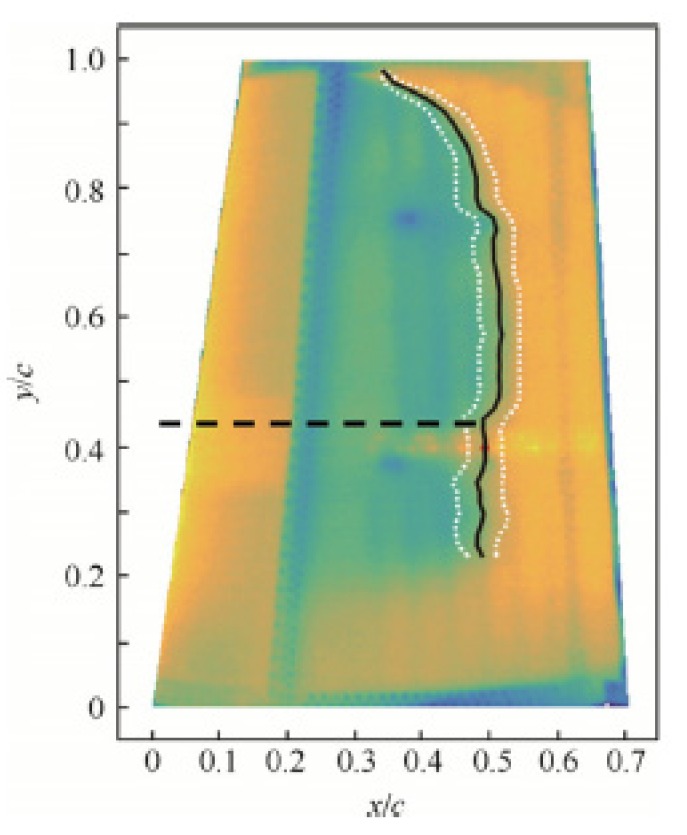
Example of infrared results for morphed wing demonstrator without aileron [34]. Figure 9 Reprinted from Chinese Journal of Aeronautics, Vol. 30 (1), Koreanski, A.; Gabor, O.S.; Acotto, J.; Brianchon, G.; Portier, G., Botez, R.M.; Mamou, M.; Mebarki, Y., Optimization and design of an aircraft’s morphing wing-tip demonstrator for drag reduction at low speed, Part. II – Experimental validation using Infra-red transition measurement from wind tunnel tests, 164–174, 2017, open access.

A novel genetic algorithm useful for the optimization of the shape and of the upper surface of an airfoil by means of actuators has been presented by Koreanski et al. [33]. The method was adopted for two dissimilar airfoils and the algorithm was related to two other optimization methods: gradient method and artificial bee colony. The outcomes of the optimization with each of method was schemed on response surfaces obtained with the Monte Carlo method. From the comparison it was demonstrated that they were placed in the global optimum region. Results for different wind tunnel test case (16) and objective Functions (2) were shown. The enhancement of about 17% of the chord for the former (transition interval) and about 31% of the chord for the latter (transition advancement) were identified. The outcomes of the performed optimization (displacement) were used as input data for the positioning control of the upper surface in the wind tunnel testing on the MDO 505 wing tip demonstrator. Comparisons between the experimental transition regions of the wing section (morphed and un-morphed) were carried out by means of infrared (IR) thermography. Numerical optimization was validated as documented in a successive paper [34] (Figure 8 and Figure 9). The optimization was accomplished for 16 flight cases by changing the following parameters: speed, attack angles, and aileron deflections. The results of the optimization of the flow for the airfoil morphing upper-surface were validated by means of IR thermography on the wing-tip demonstrator. According to the validation results, the 2D numerical optimization of the genetic algorithm was demonstrated to be a useful tool for the improvement of various aerodynamic features of a wing’s performance.

**Figure 10:**
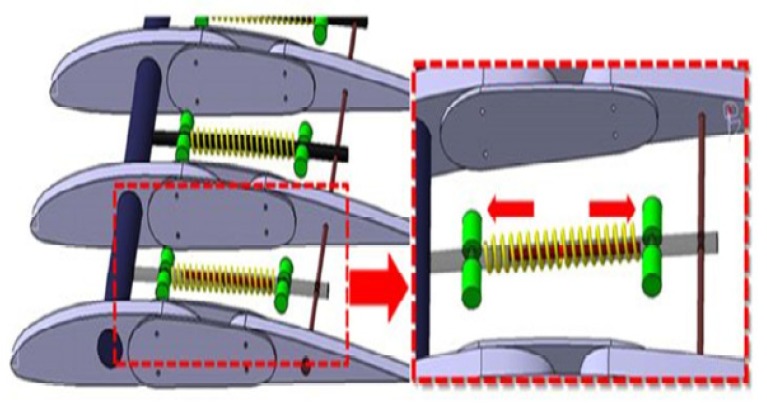
Shape memory alloy actuator (extension) in a morphing wing [35]. Figure 10 Reprinted from Materials Today Proceedings, Vol. 5, Bashir, M.; Rajendram, P.; Sharma, C.; Smrutiranjan, D., Investigation of smart material actuators & aerodynamic optimization of morphing wing, 21069–21075, 2018, with permission of Elsevier.

Also, flap can be morphed by means of shape memory alloys, as proposed and demonstrated by Bashir et al. [35]. The aerodynamic features of a flap have been examined by means of aerodynamic analyses and wind tunnel tests. The beams of the morphing flap have been manufactured with multiple elements joined together allowing rotations between adjacent elements and forming a flat shape of the morphing flap (Figure 10). Such a morphing flap consisting of multiple elements was analyzed from the aerodynamic point of view using XLFR pro: the aerodynamic behavior was compared with that of a classical mechanical-flap and the main characteristics were measured in a wind-tunnel facility. The first comparison regards the improvements of the lift increment and drag decrease of the standard mechanical flap. On the other hand, the morphing-flap wings exhibited the highest lift/drag ratio gain in the comparison with standard flap at corresponding attack angles. The comparison has shown that the maximum lift-drag ratio gains is evidenced in correspondence of the 0° angle of attack. In conclusion, if a mechanical flap is changed with a morphing flap, at 0° incident angle, a more efficient level flight can be achieved. For what concerns the results of the lift/drag ratio gain of the six-element morphing flap, the comparison where the mechanical and morphing flaps have shown their maximum lift/drag ratios was analyzed. The maximum lift/drag ratios were detected for different attack angles for the mechanical flap and for morphing flap. Results of experiments in the wind tunnel test have shown that the maximum lift/drag ratio for the morphing-flap has been detected with a 0° incident angle, independent to the flap deflection angle. The maximum efficiency of a level flight can be obtained when incident angle is set to be angle of attack of maximum efficiency. The multi-element morphing flap adopted in the final model has shown the maximum lift/drag ratio at incident angle of 0°. The experimental results of the wind tunnel test showed an increase of the lift/drag ratio of about 83.98% achieved with a flap deflection angle of 20°, which is a highly significant gain with respect to flight efficiency. Despite many studies, research projects, prototypal wind tunnel tests, and in-flight with unmanned airplane (NASA), SMA-activated morphing wings are still not adopted on modern aircrafts.

## 5. Space Applications

**Figure 11:**
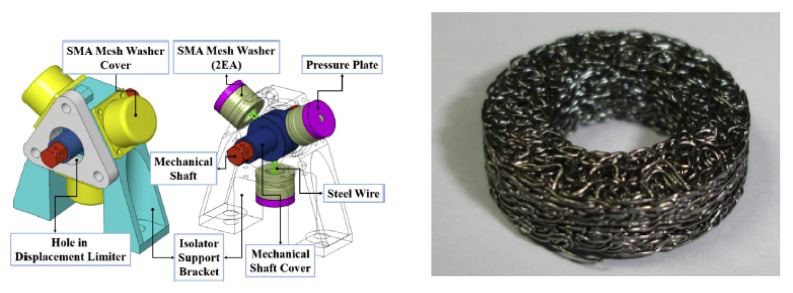
Shape memory alloys (SMA) mesh washer developed by Kwon et al. (**Left**) [36]. Pseudoelastic SMA mesh washer (**Right**) [36]. Figure 11 Reprinted from Cryogenics, Vol. 67, Kwon, S.C; Jeon, S.H.; Oh, H.U., Performance evaluation of spaceborne cryocooler micro-vibration isolation system employing pseudoelastic SMA mesh washer, 19–27, 2015, with permission of Elsevier.

Space applications of SMAs seek to solve the exceptional problems of actuation, release, and vibration attenuation in the launch of a spacecraft or subsequent operations, in conditions of microgravity and zero atmosphere. A solution to this problem has been proposed by Kwon et al. [36] with a pseudoelastic shape memory alloy mesh washer. Thanks to this device, vibration isolation is achieved in a severe launch vibration environment as well for micro-vibrations from the cryocooler on-orbit (Figure 11). Undesirable microvibrations generated by a spaceborne cryocooler during the on-orbit operation are the main source of loss of the image quality taken from high-resolution satellites. For the compliance of the mission requirement, in order to acquire high-quality images, the isolation of the micro-vibrations generated by a cryocooler is required. In the proposed solution a spaceborne cryocooler micro-vibration isolator, based on a pseudoelastic mesh washer, has guaranteed adequate vibration isolation in an environment as severe as launch. Micro-vibrations from the cryocooler on orbit have been mitigated too. Static tests and free vibration tests have been performed on the assembly and the effectiveness of the concept has been demonstrated by the micro-vibration assessment tests. The working principle of such usefully employed devices is based on the wide hysteresis and strong non-linearity exhibited during the loading and unloading cycle. An overview of the pseudoelastic behavior, modeling, and applications at the same time with their limitations in seismic isolation and vibration control can be found in the paper by Saadat et al. [37]. The evaluation of the pyroshock isolation performance of a compressed mesh washer isolators has been focused on by Youn et al. [38]. Shape memory alloy compressed mesh washer isolators have been prepared and tested. The dynamic response of the compressed mesh washer isolators due to precompressive displacements has been analyzed too. The results showed that damping capacity and the natural frequency can be modified by varying the precompressive displacement of the isolator. These features can be useful for smart isolation systems based on the adaptable dynamic capacity of the isolator.

Present solar array equipment delivers specific energies of about 20–40 W/kg in the case of the solar array deployment system. Higher specific energies, greater than 100 W/kg, can be achieved with Lightweight Flexible Solar Array (LFSA) technology proposed by Carpenter and Lyons [39]. Photovoltaic arrays could in some missions provide high power/weight ratios in comparison with conventional solar arrays. A higher science payload mass fraction can consequently be exploited. The solar array uses copper indium diselinide solar cells on a bendable substrate. Shape memory alloys applied on the hinge as a deployment system can be adopted. In this way, the heating of the SMA elements allows the hinges to be deployed [39]. In this application, the spacecraft 28 V bus can be employed. The shock-less deployment could increase the spacecraft dynamics while deployment is happening. SMA elements are also safer to be handled, integrated and tested in comparison with conventional ones. SMA hinge devices for solar array are at the same time less expensive, more reliable, and simpler. However, the development stage of SMA-activated flexible devices is still experimental.

One of the most successful uses of SMA in space is the solution to the problem of low-shock release devices. These mechanisms are very common in the design of spacecraft and have been conceived and developed some years ago, as shown by the Johnson’s Patent [40]. It deals with a release device based on non-explosive separation system for releasable components of equipment or apparatus that are required to be released under no-shock conditions. In such a separation device, the active element is strained to an armed configuration in low temperature condition that is below the activation temperature. The activation of the device happens while heating the shape memory element up to reach the transition temperature. In this way, the elements can recover its preset shape in a continuous manner, without shock. A prototype of the invention deals with a method for testing the integrity and the readiness of the low-shock separation device and at the same time a method for the calibration of the actuator’s release point. The relevance of the argument is ascribable to the fact that the pyrotechnic release mechanism has been often found as the root of failure and the cause of abortion of the mission [41]. As the activation of the SMAs happens slowly by continuous and controlled heating, SMA components can be suitably employed in low-shock release mechanisms also in micro-sized satellites. A new era is coming in device design thanks to the continuous request for satellites and spacecraft to be smaller, faster, and cheaper. Simple designs and applications based on novel technologies seem to be ideal for miniature mechanism design. The developed mechanisms in practical use include separation nut (micro and mini), mini rotary actuator, micro burn wire release, an SMA linear actuator, and an SMA redundant release mechanism [42]. Also, in small satellites, a great variety of pull-out systems to accomplish functions of many missions (separation from the launch vehicle, separation from each other, instruments deployment) are always more frequently required. Two effective flights have been arranged by the Air Force Research Laboratory on the topic of low-shock release mechanisms. The first experiment was called Shape Memory Alloy Release Device (SMARD) and it was performed in May 1999. The second experiment in January 2000 regarded the deployment of the Air Force Academy FalconSat spacecraft from the Orbital Sub-Orbital Program Space Launch Vehicle (OSP-I) [43]. Smaller rotary actuators were developed by Park et al. [44] through microfabrication methods employing shape memory wires with a small diameter (about 100 micron). It has been well established that rotary actuators of a maximum dimension of 5 mm may be constructed with an actuation angle of around +/−60°. On the basic principle of this bi-directional actuator, SMA ratchet micro mechanisms (about 200 micron) have been designed and manufactured for the transformation of angular motion in continuously rotating motion. Shape memory elements have been embedded into polyurethane structures with a suitable manufacturing process. Layer by layer in this way it is possible to manufacture complex 3D components by means of combination of material addition and subtraction. In order to offer an actuator of the same compactness with conventional systems, very small parts should be manufactured while SMA elements are at the same scale of the actuators housing itself.

**Figure 12:**
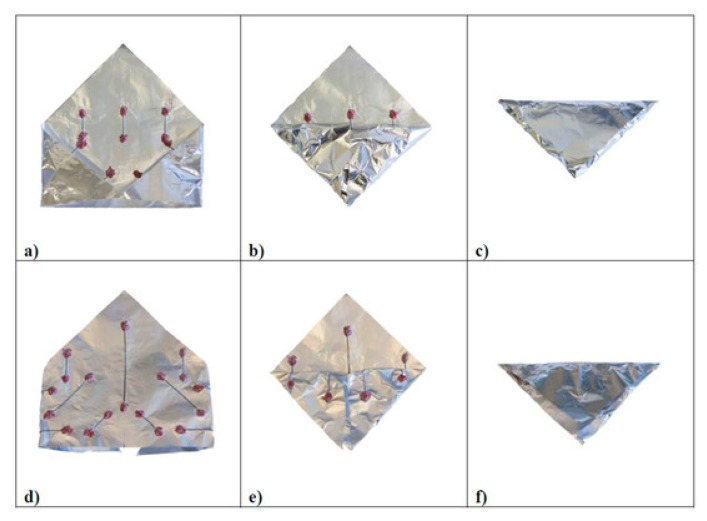
Step of solar sail folding sequence for two different configurations of the SMA actuators [47]: towards inside (**a**–**c**) and towards outside (**d**–**f**). Figure 12 Reprinted from Actuators, Vol. 8 (2), Boschetto, A.; Bottini, L.; Costanza, G.; Tata, M.E., Shape memory activated self-deployable solar sails: small scale prototypes manufacturing and planarity analysis by 3D laser scanner, Art. N. 38, 2019, open access.

**Figure 13:**
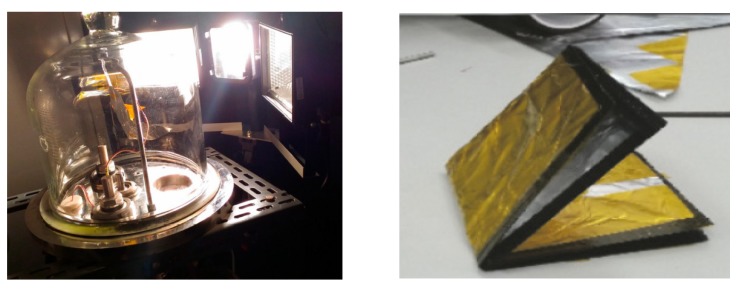
Solar-sail self-deployment experiment under vacuum condition in the bell jar (**Left**) [48], solar sail with carbon fiber on the frame integrating the SMA (**Right**) [49]. Figure 13 Reprinted from Aerospace, Vol. 6 (7), Bovesecchi, G.; Corasaniti, S.; Costanza, G.; Tata, M.E., A novel self-deployable solar sail system activated by shape memory alloys, Art. N. 78, 2019, open access e Reprinted from Advances in materials science and engineering, Vol. 2017, Costanza, G.; Leoncini, G.; Quadrini, F.; Tata, M.E. Design and characterization of a small-scale solar sail prototype by integrating NiTi SMA and carbon fibre composite, Art. N. 8467971, 2017, open access.

Another important SMA possible application field is in solar sailing, still in the small-prototype laboratory test development stage. The solar sail working principle is based on the radiation pressure acting as a propulsion system. Sunlight can be employed to move space vehicles by the reflection of solar photons from a huge and light-weight material. In this way, no propellant is necessary for primary propulsion. A novel self-deploying solar sail activated by NiTi shape memory wires has been designed and manufactured in the form of a small prototype by Costanza and Tata [45]. Aluminum and Kapton have been adopted in this experiment in order to simulate the sail. Three dissimilar arrangements have been analyzed for bending the sail. For the active elements, two NiTi wires were employed so that the self-deployment of the sail happens when the active elements approach the critical transformation temperature. In the first experiments, heating was performed by infrared lamps up to the activation of the shape memory elements. Successively, Costanza and Tata [46] focused their attention in the deployment experiments performed in the laboratory, on the analysis of different heating technique, and different pressure conditions on the activation times. By employing shape memory wires, a small solar sail prototype was self-deployed in laboratory in atmospheric conditions and in low pressure conditions (0.05 bar) just from the effect of a halogen lamp (Figure 12). In order to evaluate the effectiveness of the self-deploying system, the planarity degree of the sail has been evaluated by Boschetto et al. [47]. The level curves from the regression plane were highlighted and the differences between the four analyzed configurations analyzed. The vertices were identified as the most critical zones. Some small-scale solar sail prototypes have been studied, prepared, and tested by Bovesecchi et al. [48] in three different environmental conditions to simulate, as much as possible, the real working environment of solar sails (Figure 13). In the same work, heat transfer mechanisms were analyzed too. Moreover, to demonstrate the feasibility of the project, the smallest distance from the sun allowing the full activation of the SMA elements and the self-deployment of the sail was also calculated. The integration of SMAs with carbon fiber composite in a solar sail was discussed for the optimization of the surface/weight ratio by Costanza et al. [49]. In a device conceived for one-time activation (i.e., micro-actuator for satellite), the training of the SMA is not required and the shape memory stability is not a problem. But if the system is designed for many activation cycles, the stability of the material, the capability to perform many cycles in a stable manner, as well fatigue behavior (mechanical, thermal, or combination or both) must be considered. The capabilities of SMA at a high number of activation cycles has been focused on and discussed in many papers. Shape memory alloys can successfully be employed to provide linear displacements. Costanza et al. [14] demonstrated that SMA springs can be subjected to up to 600,000 activation cycles with a limited and controlled loss of the shape memory recovery. The stabilization of the SMA spring’s behavior with the increase of the working cycle has occurred as evidenced in [14]. The loss of the shape recovery efficiency has been quantified in [15]. The phenomenon is ascribable to functional fatigue and the formation of the R phase upon heating has been evidenced by resistivity measurements. The effect of temperature on the tensile behavior of NiTi sheets has been discussed in [50]. Four NiTi flat elements have been analyzed in tensile conditions (load/unload cycles) at constant temperatures from room temperature up to 60 °C. The shape recovery has been measured and was found to be higher at increasing testing temperatures. The energy dissipation on each load-unload cycle has been measured and found to be greater at higher temperatures.

A prototypal linear actuator activated by shape memory elements with modular stroke has been designed and manufactured by Costanza et al. [51]. The developed prototype has been employed for the analysis of the working principle and its response with particular reference to the positioning and the displacement. The opening and closing of the actuator is performed by the Joule effect. One of the main innovations of the proposed prototype is the possibility to modulate opening and closing just by acting on the corresponding SMA springs. As the applied voltage is changed, the opening or closing springs can be activated in order to set the desired positioning and overcome the limits of the standard configuration SMA spring—steel spring (bias).

## 6. Conclusions and Future Trend

In the last two decades, many applications of SMAs in the aerospace field have been identified, designed, modeled, and tested. Many others will be conceived and developed in the near future. The present review confirms that SMAs attracted researcher’s attention especially in the applications in which the added value is more important than the pure economic saving. Extensive studies, results of analytical and numerical modeling, and experimental tests have been found in literature regarding SMA. In many cases, the SMAs play the role of an actuator. Starting from the basic principle and the mechanical behavior (shape memory effect and pseudoelastic), many applications regarding the wing morphing have been presented (experimental and modeling) as well tailoring of the inlet geometry of various propulsion systems. Variable geometry chevron actuated by SMA has been illustrated for thrust, noise, and general efficiency optimization. Also, space applications have been described for isolating the micro-vibrations, for low-shock release devices and self-deployable solar sails. The development stage in many cases is limited to prototypes tested in laboratories or in wind tunnels, with only a few actually flown and even fewer in use. The main limitations may be ascribable to the higher weight, in some cases complexity of the systems, and the structural and functional fatigue phenomena in cycling applications. In the near future, many other SMA-based applications will be identified and conceived in which the actuation due to the shape memory effect or the pseudoelasticity will be based on.
